# Genome-wide identification and characterization of NAC transcription factor family members in *Trifolium pratense* and expression analysis under lead stress

**DOI:** 10.1186/s12864-023-09944-8

**Published:** 2024-01-31

**Authors:** Zicheng Wang, Zirui Chen, Yuchen Wu, Meiqi Mu, Jingwen Jiang, Wanting Nie, Siwen Zhao, Guowen Cui, Xiujie Yin

**Affiliations:** Department of Grassland Science, College of Animal Science and Technology, Northeast Agricultural University, Harbin, 150030 China

**Keywords:** Pb stress, *NAC*, Gene family, Red clover, qRT-PCR

## Abstract

**Background:**

The NAC TF family is widely involved in plant responses to various types of stress. Red clover (*Trifolium pratense*) is a high-quality legume, and the study of *NAC* genes in red clover has not been comprehensive. The aim of this study was to analyze the NAC gene family of red clover at the whole-genome level and explore its potential role in the Pb stress response.

**Results:**

In this study, 72 *TpNAC* genes were identified from red clover; collinearity analysis showed that there were 5 pairs of large fragment replicators of *TpNAC* genes, and red clover was found to be closely related to *Medicago truncatula*. Interestingly, the *TpNAC* genes have more homologs in *Arabidopsis thaliana* than in soybean (*Glycine max*). There are many elements in the *TpNAC* genes promoters that respond to stress. Gene expression analysis showed that all the *TpNAC* genes responded to Pb stress. qRT-PCR showed that the expression levels of *TpNAC29* and *TpNAC42* were significantly decreased after Pb stress. Protein interaction network analysis showed that 21 *TpNACs* and 23 other genes participated in the interaction. In addition, the TpNAC proteins had three possible 3D structures, and the secondary structure of these proteins were mainly of other types. These results indicated that most *TpNAC* members were involved in the regulation of Pb stress in red clover.

**Conclusion:**

These results suggest that most *TpNAC* members are involved in the regulation of Pb stress in red clover. *TpNAC* members play an important role in the response of red clover to Pb stress.

## Introduction

In the natural environment, drought stress, salt stress, heavy metal stress, extreme temperature stress and other abiotic stresses hinder the normal growth and development of plants, resulting in a decline in plant yield and ecological economic value [1, 2]. The impact of heavy metal stress on plants is becoming increasingly serious. Heavy metal elements such as lead (Pb), mercury (Hg) and cadmium (Cd) enter the environment in large quantities via human activities and are enriched in the food chain, which intensifies heavy metal stress on plants and threatens human health [3]. There have been some studies on plant resistance to heavy metal stress, for example, studies on metal tolerance proteins (MTPs) [4], ATP-binding box (ABC) transporters [5], natural resistance-associated macrophage proteins (NRAMPs) and oligopeptides (OPTs) in plants that are responsive to heavy metal stress [6, 7]. However, the gene regulatory network involved in plant resistance to heavy metal stress remains unclear. Lead is a naturally occurring bivalent trace metal element that readily accumulates in plants. High concentrations of Pb inhibit plant growth and development, weaken plant photosynthesis, and have toxic effects on plant cells. Transcriptional regulation is one of the important mechanisms of plant resistance to lead stress. *PSE1* has been reported to significantly improve lead tolerance in plants by inducing phytochelatin (PC) synthesis and activating the expression of genes related to PC synthesis, increasing the accumulation of glutathione (GSH) and PCs [8–10].

NAC TFs are widely found in plants, and their N-terminal domain is a highly conserved NAC domain that binds to DNA. This domain was first identified at the N-terminus of the protein encoded by the *NAM* gene of *Petunia hybrida* [11–13]. Subsequently, similar conserved domains were found at the N-terminus of *A. thaliana* ATAF1/2 and CUC2, hence the name NAC domain. The NAC domain can be further divided into 5 subdomains: A, B, C, D and E. A, C and D are highly conserved subdomains [14]. A participates in the formation of functional dimers; C and D are DNA-binding sites; and B and E have variability, which is related to the functional diversity of NAC TFs. The C-terminus of NAC TFs is a transcriptional regulatory region (TRR) with high variability [15, 16].

NAC TFs are involved in plant resistance to abiotic stress. Overexpression of *ANAC019*, *ANAC055* and *ANAC072* in *A. thaliana* can improve drought resistance [17]. The *SNAC1* gene is related to salt tolerance and drought tolerance in rice. The *TaNAC2* and *TaNAC67* genes affect the salt tolerance, drought resistance and cold resistance of *A. thaliana* and *T. aestivum* [18, 19]. The expression levels of 19 *SlNAC* genes in tomato (*Solanum lycopersicum*) changed significantly under aluminum (Al) stress, and 5 NAC TFs in kenaf were responsive to lead stress [20]. NAC TFs have been identified at the genomic level in an increasing number of species; for example, 105, 152, and 93 *NAC* genes have been identified in *A. thaliana*, soybean, and tomato, respectively, but until now, the *NAC* gene in red clover has been poorly studied [20–22].

Red clover, belonging to a genus of legumes, is a perennial herb native to Asia Minor and Southeastern Europe. It is an important forage with high nutritional value, a fast growth rate, and beneficial nitrogen fixation and soil quality improvement abilities. The planting and cultivation of red clover are often affected by abiotic stresses such as cold, drought and heavy metal stress [23, 24]. Through transcriptomic and metabolomic analysis, Meng et al. revealed the mechanism underlying the response of red clover to different concentrations of Pb stress and found that under low concentrations of Pb (500 mg/kg), the three pathways of “carbon metabolism”, “glycine and dicarboxylic acid metabolism” and “amino acid biosynthesis” exhibited significant responses. A high concentration of Pb (3000 mg/kg) influenced the “hormone signal transduction” and “starch and sucrose metabolism” pathways in the plants. In addition, TFs such as C2H2, AP2/ERF-ERF, bHLH, MYB, FAR1, WRKY and NAC in red clover leaves exhibited positive responses to Pb stress [25].

In this study, 72 *TpNAC* genes were identified from the red clover genome, and these *TpNAC* genes were mapped to chromosomes. Then, phylogenetic analysis, collinearity analysis, gene structure and motif analysis, and Cis‑element analysis were performed. Based on the results published by Meng et al., *TpNAC* genes responding to Pb stress were selected for qRT‒PCR verification [25]. The interaction network and a three-dimensional structure model of the proteins encoded by the *TpNAC* genes were predicted. Analysis of *TpNAC* gene expression patterns showed that most *TpNAC* genes were specifically expressed under Pb stress. Further study of these specifically expressed *TpNAC* genes will help elucidate the adaptive and resistance mechanisms of red clover. In summary, the results of this study will help in further study of the role of TpNAC TFs in the response of red clover to Pb stress and provide new information for molecular breeding of stress-resistant red clover.

## Result

### Identification and protein characterization of the TpNAC gene family

Using HMMER 3.0 software, 94 NAC sequences were found in the red clover database based on the presence of the NAM conserved domain (Pfam: PF02365). After removing incomplete NAM domain sequences and submitting them to Pfam for verification, 72 TpNAC TFs were finally identified and named *TpNAC1*-*TpNAC72* according to their distribution on chromosomes **(Table S2)**. The physicochemical properties of the encoded proteins were analyzed. The amino acid sequence length of the encoded proteins ranged from 146 to 1803, the pI ranged from 4.56 to 10, and the molecular weight ranged from 1717.88 to 202280.84 Da **(**Table 1**)**. In the instability coefficient range of 22.99 to 62.52, there were 30 *TpNAC* genes encoding stable proteins and 36 encoding unstable proteins. The number of *TpNAC* genes encoding unstable proteins was slightly higher than that encoding stable proteins. The adipose index ranged from 46.1 to 84.29. The total mean hydrophilic range was − 1.06~ -0.323, and all the proteins encoded by *TpNAC* genes were hydrophilic proteins **(**Table 1**)**. Subcellular localization prediction results showed that most *TpNAC* genes were localized in the nucleus, and five *TpNAC* genes (*TpNAC7*, *TpNAC17*, *TpNAC29*, *TpNAC30*, and *TpNAC38*) were localized in the cytoplasm. Two *TpNAC* genes (*TpNAC2* and *TpNAC62*) were localized in the extracellular system, and three *TpNAC* genes (*TpNAC47*, *TpNAC49* and *TpNAC56*) were localized in the mitochondria. Four *TpNAC* genes (*TpNAC8*, *TpNAC14*, *TpNAC19* and *TpNAC66*) were localized on the plasma membrane **(**Table 1**)**. In this study, differences in amino acid sequence length and other physical and chemical properties of the proteins encoded by *TpNAC* genes were observed. The wide ranges of the indices indicated that the TpNAC proteins have different biochemical properties and functions and play roles in different locations in cells.

**Table 1:** Identification of basic physical and chemical properties of TpNAC gene family members and prediction of subcellular localization

Gene Name	Amino Acid(bp)	Mass(Da)	pI	Instability Index	Fat Index	Average Hydropathicity	Subcellular localization
TpNAC1	374	42774.89	5.65	34.41	64.39	-0.752	Nuclear
TpNAC2	272	31311.4	5.74	22.99	71.29	-0.488	Extracellular
TpNAC3	338	38831.69	5.2	48.91	76.09	-0.534	Nuclear
TpNAC5	296	33899.99	8.94	52	72.8	-0.604	Nuclear
TpNAC6	360	41408.45	5.27	51.19	67.69	-0.7	Nuclear
TpNAC7	266	29852.81	6.63	38.96	71.05	-0.657	Cytoplasmic
TpNAC8	223	25750.16	5.79	29.31	80.36	-0.394	Plasma Membrane
TpNAC9	465	52306.9	6.06	44.85	59.81	-0.802	Nuclear
TpNAC10	623	71506.54	5.22	44.67	70.05	-0.611	Nuclear
TpNAC11	263	30150.4	6.38	38.4	61.9	-0.636	Nuclear
TpNAC12	349	40702.21	6.24	44.98	59.26	-0.924	Nuclear
TpNAC13	494	55580.25	6.68	51.17	56.62	-0.981	Nuclear
TpNAC14	405	47116.5	8.14	36.13	63.06	-0.572	Plasma Membrane
TpNAC15	1803	202280.84	5.18	49.81	70.64	-0.542	Nuclear
TpNAC16	186	21596.7	4.56	49.97	66.99	-0.742	Nuclear
TpNAC17	288	33499.77	5.85	30.46	59.9	-0.781	Cytoplasmic
TpNAC18	302	34461.07	9	29.95	67.42	-0.627	Nuclear
TpNAC19	287	32973.59	9.11	39.67	63.48	-0.438	Plasma Membrane
TpNAC20	374	41112.82	4.96	46.11	70.11	-0.356	Nuclear
TpNAC21	594	66541.7	5.27	44.37	68.47	-0.662	Nuclear
TpNAC22	349	40447.37	6.05	38.69	61.72	-0.823	Nuclear
TpNAC24	336	39177.49	6.68	51.17	72.53	-0.642	Nuclear
TpNAC25	261	30077.75	4.91	56.11	60.5	-0.63	Nuclear
TpNAC27	291	33580.55	8.32	41.78	63.26	-0.723	Nuclear
TpNAC28	456	52095.44	7.64	31.22	74.19	-0.435	Nuclear
TpNAC29	147	17171.88	9.08	38.74	76.26	-0.427	Cytoplasmic
TpNAC30	150	17787.22	9.52	27.57	63	-0.916	Cytoplasmic
TpNAC31	382	44721.81	6.32	42.86	49.19	-1.06	Nuclear
TpNAC32	444	49717.95	6.08	46.93	62.18	-0.76	Nuclear
TpNAC33	392	43742.25	7.06	25.42	84.26	-0.468	Nuclear
TpNAC34	270	31221.26	7.63	62.52	58.11	-0.817	Nuclear
TpNAC35	482	53,831	4.86	41.27	76.1	-0.577	Nuclear
TpNAC36	396	45,656	8.19	33.54	59.8	-0.924	Nuclear
TpNAC37	346	39166.24	9.12	40.03	70.78	-0.666	Nuclear
TpNAC38	193	22884.54	9.4	37.3	76.68	-0.396	Cytoplasmic
TpNAC39	342	39037.91	6.97	33.19	61.55	-0.662	Nuclear
TpNAC40	314	35669.04	7.79	59.74	58.34	-0.75	Nuclear
TpNAC41	484	55463.73	8.18	45.52	64.26	-0.584	Nuclear
TpNAC42	202	23810.14	9.42	35.86	54.95	-0.944	Nuclear
TpNAC43	364	41226.16	8.16	27.54	64.75	-0.655	Nuclear
TpNAC44	396	44406.61	9.21	40.25	58.86	-0.793	Nuclear
TpNAC45	430	49358.06	6.61	42.24	72.3	-0.429	Nuclear
TpNAC47	266	31617.21	10.0	33.56	84.29	-0.323	Mitochondrial
TpNAC48	314	35779.64	8.82	42.31	61.78	-0.629	Nuclear
TpNAC49	189	22358.35	9.57	23.54	55.24	-0.808	Mitochondrial
TpNAC50	285	32772.89	8.38	37.59	64.21	-0.866	Nuclear
TpNAC51	386	44787.1	5.88	48.97	66.14	-0.839	Nuclear
TpNAC53	514	57810.81	6.97	46.41	66.4	-0.684	Nuclear
TpNAC54	360	41013.59	5.37	42.97	55.75	-0.711	Nuclear
TpNAC55	588	66,826	5.15	57.16	77.55	-0.612	Nuclear
TpNAC56	215	24810.18	9.61	39.4	63.53	-0.69	Mitochondrial
TpNAC57	299	34503.25	5.78	43.77	73.01	-0.633	Nuclear
TpNAC58	341	39658.03	5.96	38.86	58.89	-0.862	Nuclear
TpNAC59	177	20482.33	9.12	37.27	69.32	-0.753	Nuclear
TpNAC60	352	40056.9	8.71	34.48	64.52	-0.75	Nuclear
TpNAC61	146	17300.62	9.49	33.15	46.1	-1.051	Nuclear
TpNAC62	300	34826.41	6.25	29.02	64.63	-0.614	Extracellular
TpNAC63	352	39739.56	6.21	42.46	67.47	-0.759	Nuclear
TpNAC64	195	22722.06	4.87	56.6	60.41	-0.905	Nuclear
TpNAC65	470	53719.8	6.38	30.77	72.81	-0.493	Nuclear
TpNAC66	363	41974.98	5.05	46.73	62.84	-0.58	Plasma Membrane
TpNAC68	524	59451.24	4.75	52.39	63.26	-0.474	Nuclear
TpNAC69	322	37400.81	6.56	37.6	61.15	-0.842	Nuclear
TpNAC70	411	46641.48	5.92	44.32	78.52	-0.668	Nuclear
TpNAC71	329	37488.17	6.37	40.36	62.8	-0.63	Nuclear
TpNAC72	407	47305.91	4.91	43.33	63.71	-0.634	Nuclear

**Fig. 1:**
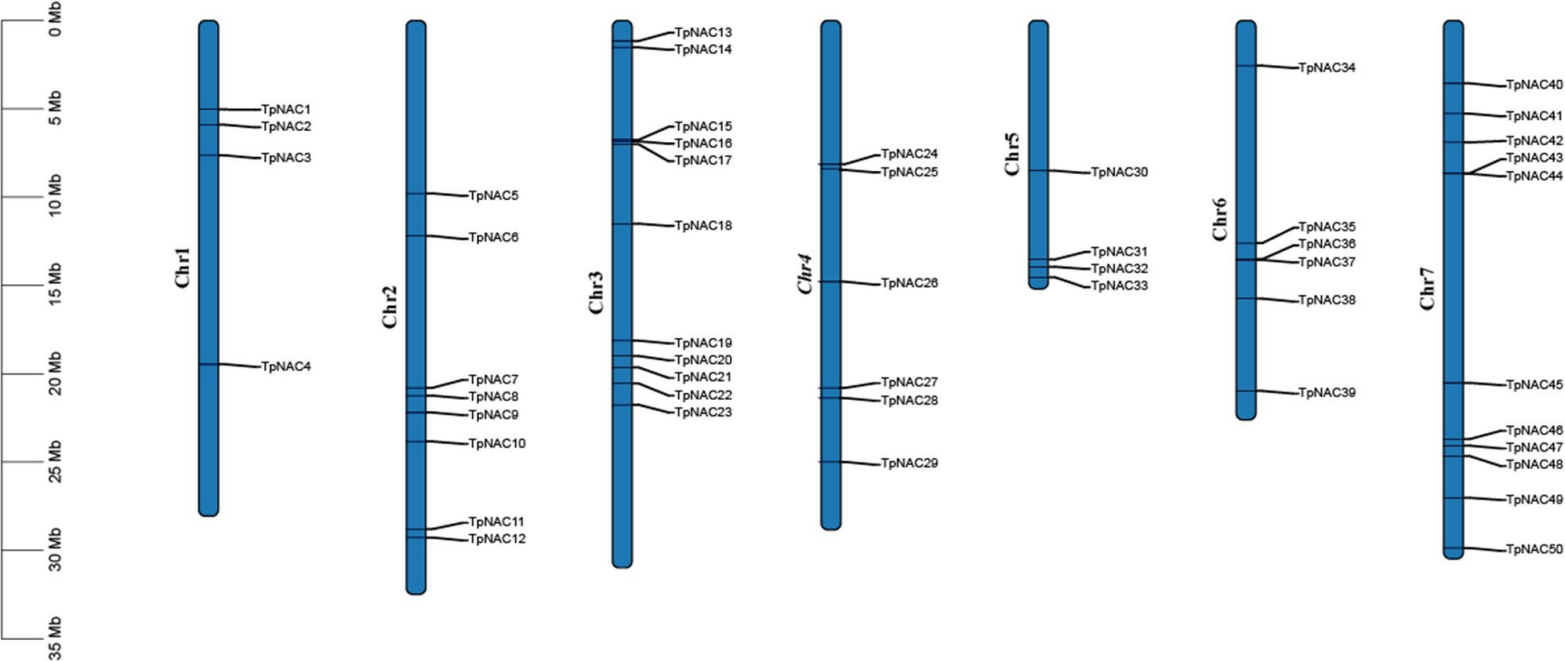
Distribution of *TpNAC* genes in the red clover chromosome. The length of each chromosome is denoted in Mbs

Chromosomal localization analysis showed that there were 50 *TpNAC* genes distributed on 7 chromosomes of red clover leaves, but the distribution was uneven. There were at most 11 *TpNAC* genes distributed on Chr3 and Chr7, and there were 4, 8, 6, 4 and 7 *TpNAC* genes distributed on Chr1, Chr2, Chr4, Chr5 and Chr6, respectively (Fig. 1).

### Classification and phylogenetic relationships of *TpNACs*

**Fig. 2:**
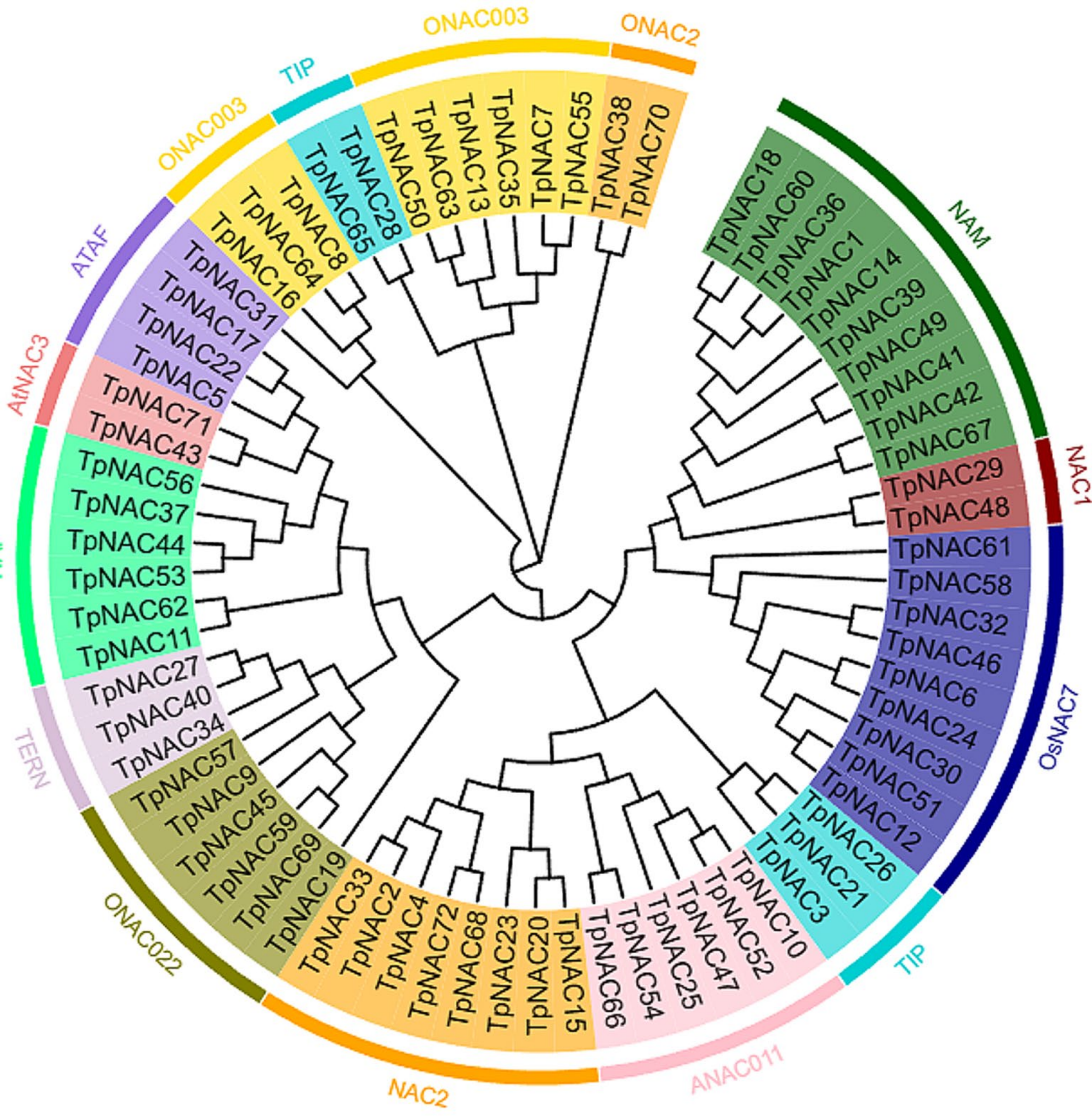
Phylogenetic cluster analysis of 72 identified NAC proteins from red clover. Different colors represent different subfamilies

The phylogenetic evolutionary tree of 72 *TpNAC* genes was constructed. The results showed that the 72 *TpNAC* genes could be divided into 13 subfamilies (Fig. 2). The NAM and NAC2 subfamilies had the most *TpNAC* genes with 10, followed by the *ONAC003* and *OsNAC7* subfamilies with 9 *TpNAC* genes. The ANAC011, NAP and ONAC022 subfamilies contained 6 *TpNAC* genes. The TIP subfamily contained 5 *TpNAC* genes; the ATAF subfamily contained 4 *TpNAC* genes; the TERN subfamily contained 3 *TpNAC* genes; and the AtNAC3 and NAC1 subfamilies contained 2 *TpNAC* genes.

### Collinearity and evolution analysis of *TpNACs*

**Fig. 3:**
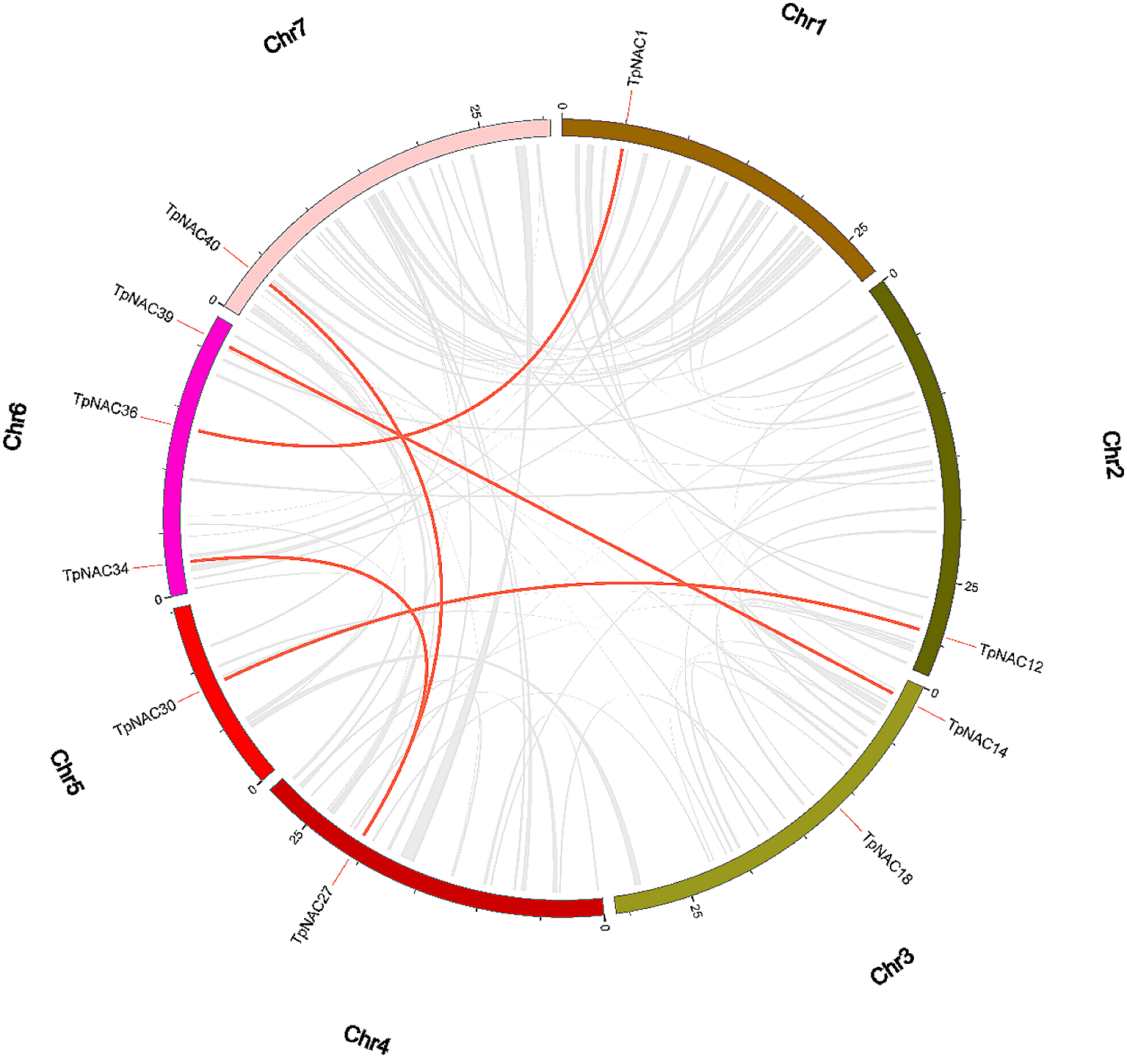
Syntenic relationship of *TpNAC* genes. The 72 *TpNAC* genes are labeled according to their chromosomal distribution in the lotus genome, and the large replications are linked by red lines

The collinearity analysis results showed that there were 5 pairs of large fragment replications between *TpNAC* genes in red clover (Fig. 3): *TpNAC1*-*TpNAC36*, *TpNAC12*-*TpNAC30*, *TpNAC14*-*TpNAC39*, *TpNAC27*-*TpNAC34* and *TpNAC27*-*TpNAC40*. All genes with large fragment replications belonged to the TERN, NAM and OsNAC7 subfamilies. There were 2 pairs of large fragment replication genes in subfamilies TERN and NAM and 1 pair in subfamily OsNAC7. Large fragment replication occured within the same subfamily.

**Fig. 4:**
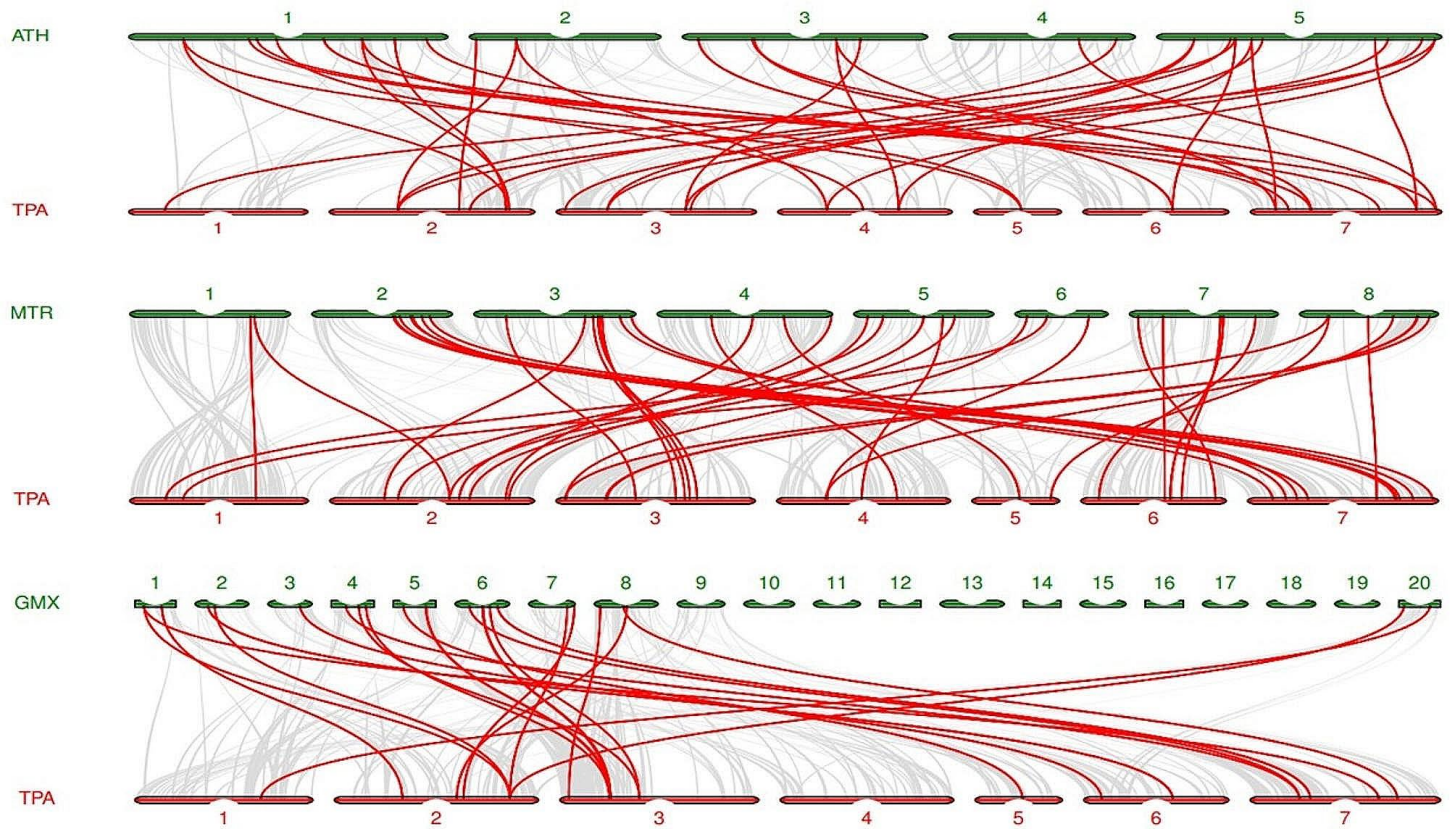
Collinearity analysis of *NAC* genes in red clover and three representative plants. The gray lines in the background represent collinear block plant genomes of red clover and other plants, and the red lines represent homologous *NAC* gene pairs. ATH represents *Arabidopsis thaliana*, TPA represents red clover, and MTR represents *Medicago truncatula*

To understand the evolution of the TpNAC gene family, the homology of TpNAC gene family members among different species was analyzed in *A. thaliana*, *M. truncatula* and *G. max*. Forty-three pairs of homologous genes were identified between *M. truncatula* and *T. pratense*, and only one *TpNAC* gene had two homologs in *M. truncatula*. Thirty-seven pairs of homologous genes were identified in *T. pratense* and *A. thaliana*; 11 *TpNAC* genes had multiple homologs in *A. thaliana*, among which *TpNAC7* had 3 homologs, and the other *TpNAC* genes had 2 homologs. Twenty-six pairs of base homologous genes were identified in *T. pratense* and *G. max*, among which five *TpNAC* genes had multiple homologs in *G. max*, including four homologs of *TpNAC10*, five homologs of *TpNAC19*, and 2 homologs of other *TpNAC* genes (Fig. 4). The above results show that *T. pratense* is closely related to *M. truncatula*. Interestingly, the number of *TpNAC* homologs in *A. thaliana* was higher than that in *G. max*, another legume. In addition, 11 *TpNAC* genes had homologs in *A. thaliana*, *M. truncatula* and *G. max*, so it was concluded that multiple replication events of *NAC* genes occurred during the evolution of these species. The eleven *TpNAC* genes with homologs in *A. thaliana*, *M. truncatula* and *G. max* played an important role in the evolution of *TpNAC* genes.

### Motifs and gene structure of TpNAC

**Fig. 5:**
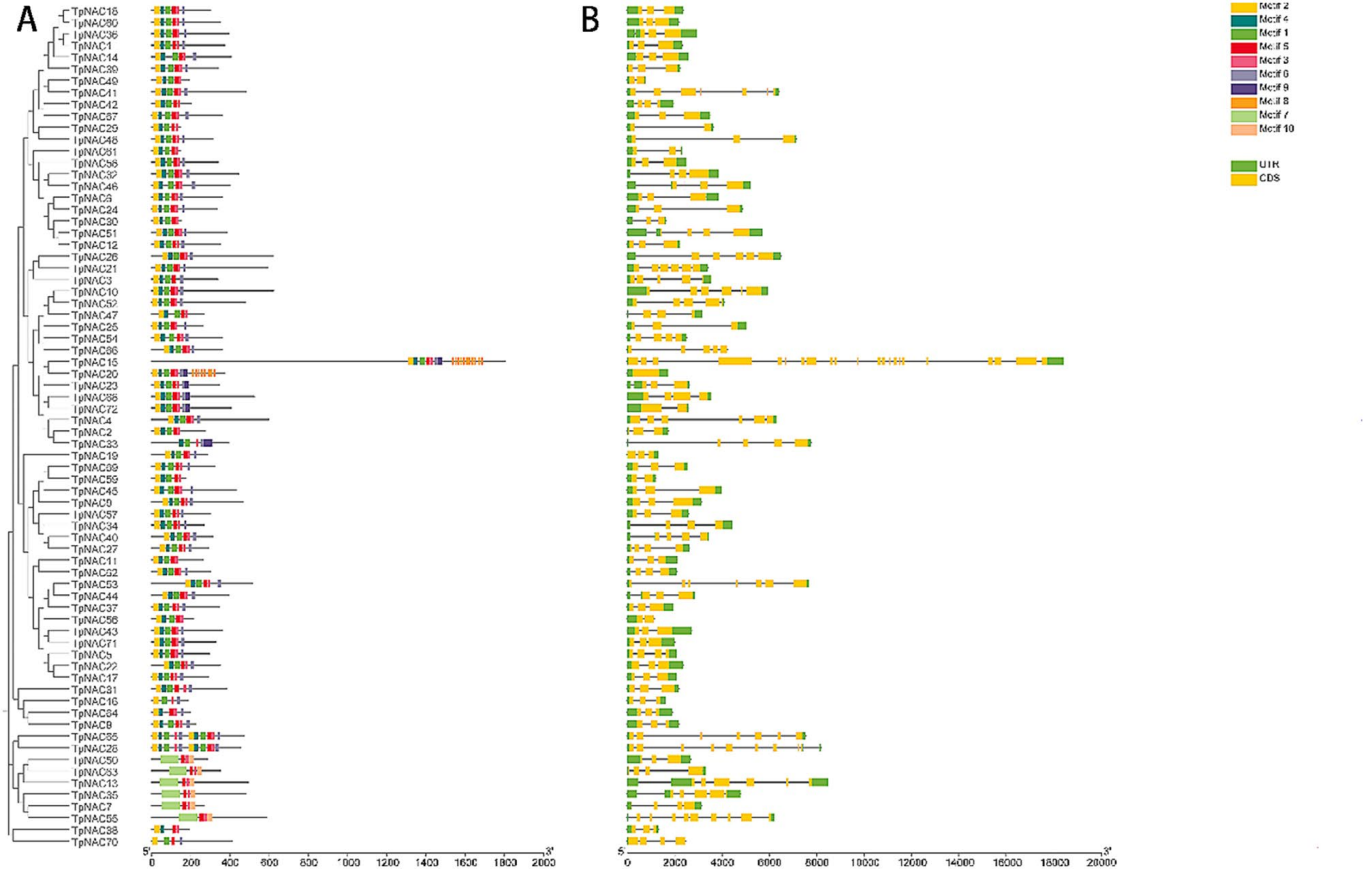
Motif and *TpNAC* gene structure analysis. (**A**) Motif analysis of NACs in red clover. (**B**) *NAC* gene structure in red clover

Ten motifs were identified by motif analysis of protein sequences encoded by *TpNAC* genes. Motif 3 and Motif 5 were found in all TpNAC proteins, and Motif 4 and Motif 7 were found in TpNAC70 and TpNAC64. Motif 9 was found in all TpNACs except TpNAC2 and TpNAC4 in subfamily NAC2. Among all the proteins encoded by *TpNAC* genes, only TpNAC8, TpNAC64 and TpNAC16 in subfamily ONAC003 contained Motif 7 (Fig. 5A).

The structure of *TpNAC* genes was visualized. The results showed that *TpNAC15* had only one exon, and the other *TpNAC* genes had more than two exons. The motif distribution and gene structure within the same subfamily were similar, so genes within the same subfamily were functionally related (Fig. 5B).

### Cis‑element analysis of the TpNAC gene family

**Fig. 6:**
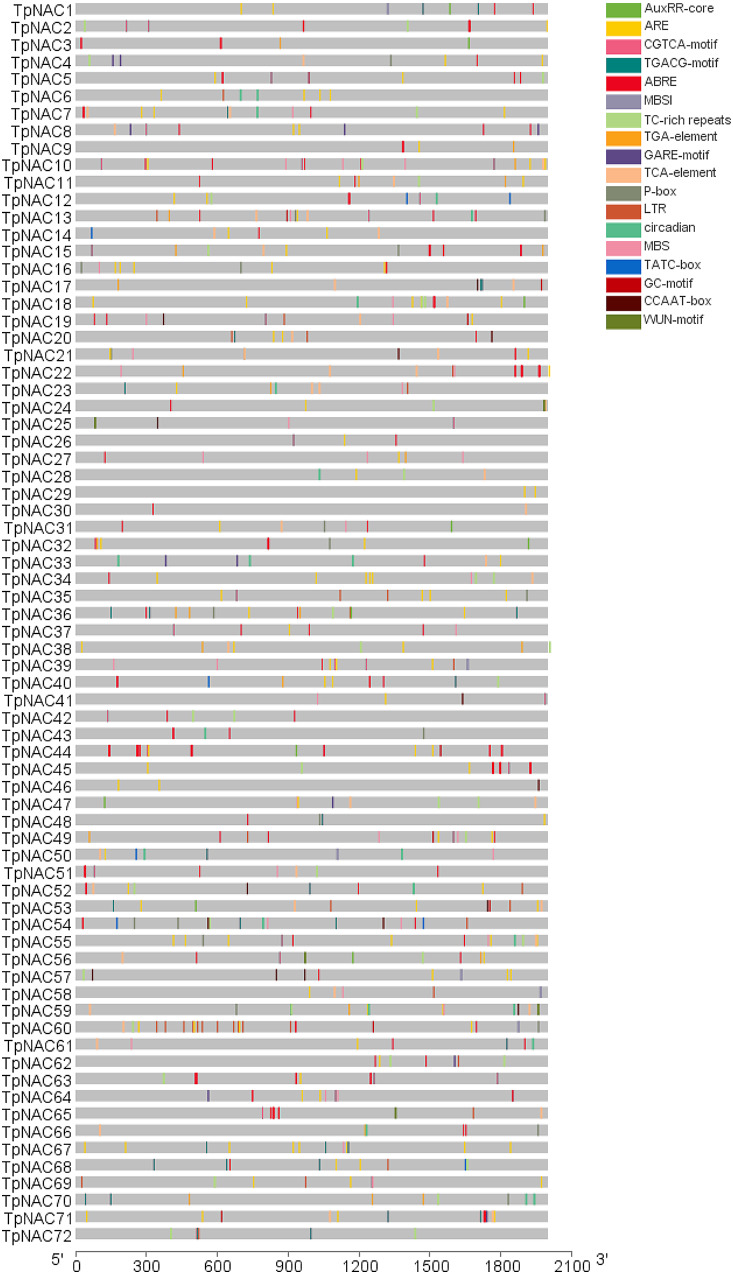
Analysis of cis-elements of TpNAC TFs. Cis-elements involving different environmental stress responses or responses to different hormones are given different colors

The 2 kb sequences upstream of the CDS of the TpNAC gene family were intercepted for homeogenic element analysis, and many cis-elements, including ARE, TC-rich, LTR, CGTCA-motif, TGACG-motif, etc., related to anaerobic reactions, defense and abiotic stress, and methyl jasmonate reactions, were identified (Fig. 6). We speculate that the TpNAC TFs are widely involved in the response of red clover to different abiotic and biological stresses and may have many potential functions in improving the stress resistance of red clover.

### Expression patterns of *TpNAC* genes under pb stress

**Fig. 7:**
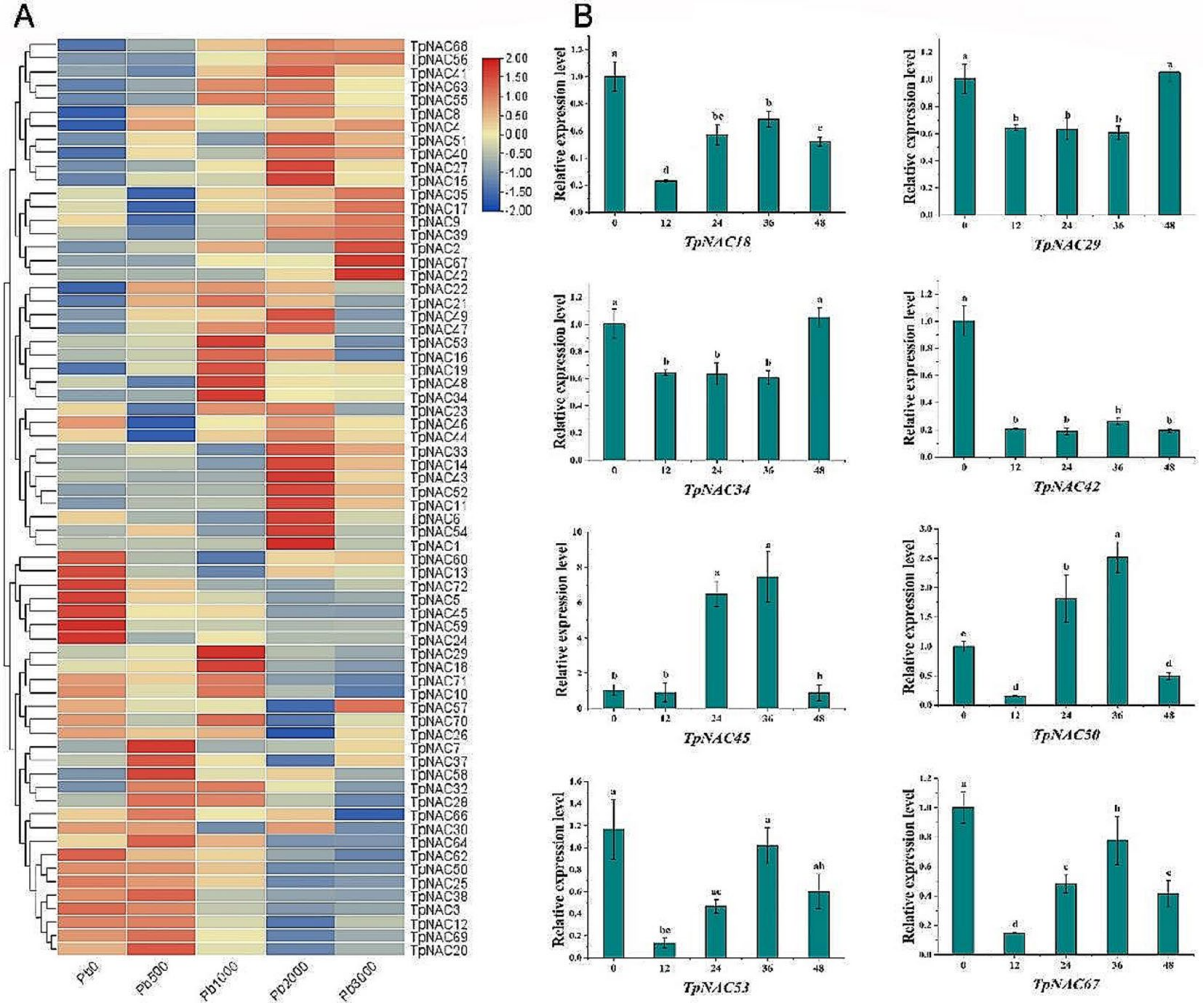
Analysis of the expression patterns of *TpNACs* under different concentrations of lead stress. (**A**) Expression calorigram of *TpNAC* genes after 45 days of lead stress treatment at concentrations of 0, 500, 1000, 2000 and 3000 mg/kg. (**B**) The expression levels of 8 *TpNAC* genes were measured at 0 h, 12 h, 24 h, 36 and 48 h in 45-day-old red clover leaves subjected to 1000 mg/kg stress

Under different levels of Pb stress, the *TpNAC* genes all exhibited different levels of response to the stress, and the expression of 5 *TpNAC* genes (*TpNAC39*, *TpNAC42*, *TpNAC56*, *TpNAC67*, and *TpNAC68*) increased with increasing Pb concentration. The expression levels of 9 *TpNAC* genes (*TpNAC3*, *TpNAC5*, *TpNAC25*, *TpNAC38*, *TpNAC45*, *TpNAC50*, *TpNAC59*, *TpNAC62*, and *TpNAC72*) decreased with increasing Pb concentration. The expression of 3 *TpNAC* genes (*TpNAC7*, *TpNAC37*, and *TpNAC58*) peaked under 500 mg/kg lead stress. There were 6 *TpNAC* genes (*TpNAC18*, *TpNAC19*, *TpNAC29*, *TpNAC34*, *TpNAC48*, and *TpNAC53*) whose expression peaked under 1000 mg/kg lead stress. There were 9 *TpNAC* genes (*TpNAC1*, *TpNAC6*, *TpNAC11*, *TpNAC15*, *TpNAC27*, *TpNAC43*, *TpNAC49*, *TpNAC52*, and *TpNAC54*) whose expression levels peaked under 2000 mg/kg lead stress. These results indicate that different *TpNAC* genes had different responses to lead stress (Fig. 7A).

It is concluded that TpNAC plays an important role in the regulation of plant resistance to lead. To study the expression of NAC TFs under lead stress, eight *TpNAC* genes were selected according to the transcriptome sequencing results (Fig. 7A), and the changes in their transcription abundance under lead stress were analyzed by qRT‒PCR. The expression levels of *TpNAC18*, *TpNAC29* and *TpNAC42* under lead stress decreased significantly and were always lower than those without lead stress. The expression of *TpNAC42* under stress was much lower than that without stress. Although the expression level of *TpNAC29* under stress was consistently lower than that without stress, it increased gradually from 12 to 36 h and decreased again at 48 h. The expression of *TpNAC34* from 12 to 36 h under stress was lower than that at 48 h without stress. The expression levels of *TpNAC45* and *TpNAC50* after 24 and 36 h of stress were significantly higher than those at other times. The expression levels of *TpNAC53* and *TpNAC67* decreased significantly after 12 h of stress and then gradually increased and decreased after 48 h, and the expression levels of *TpNAC53* and *TpNAC67* under stress were lower than those without stress. The results showed that *TpNAC29* and *TpNAC42* had the most evident response.

### Protein interaction network prediction for TpNACs

**Fig. 8:**
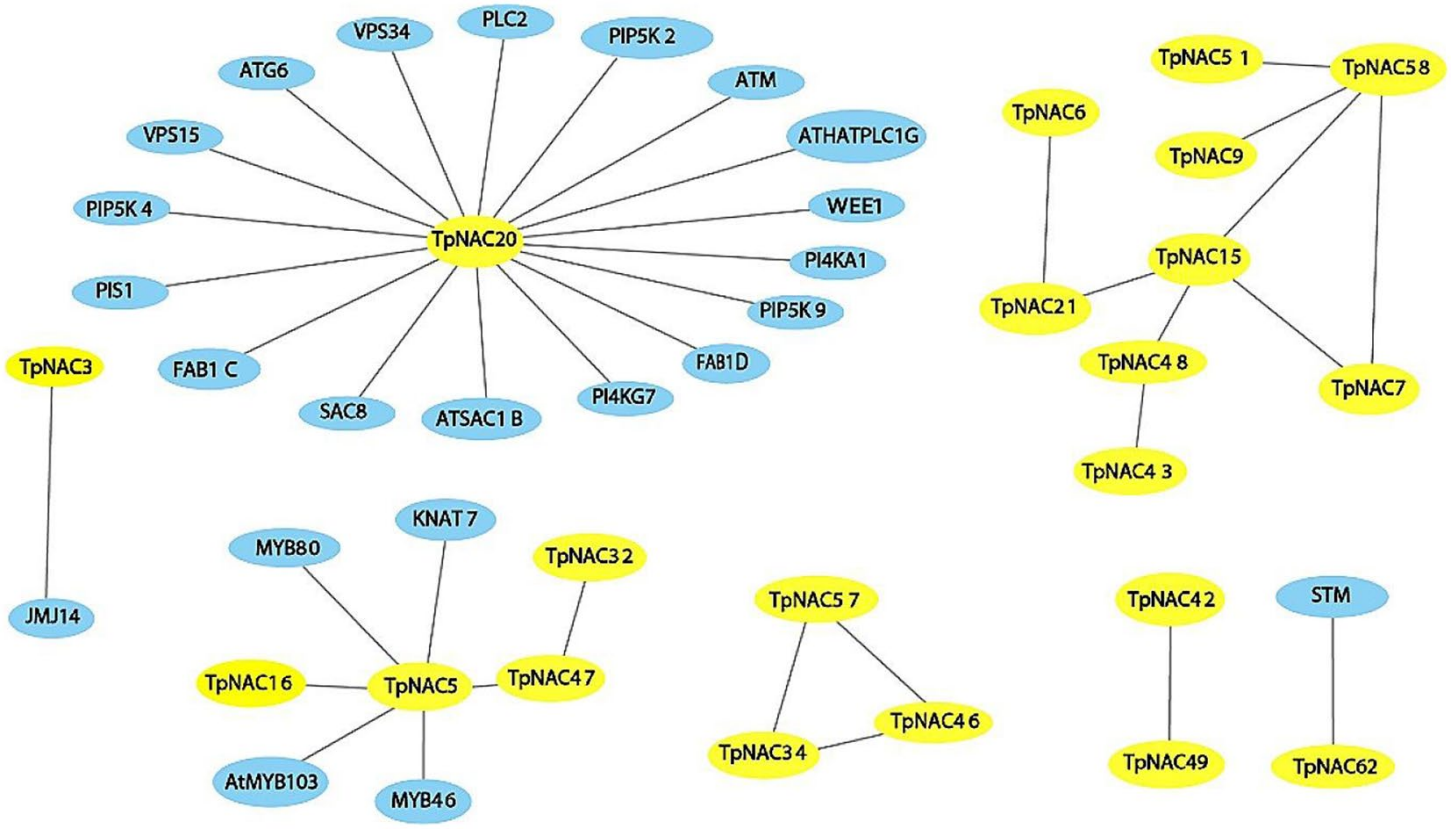
Protein interaction networks illustrating the associations between different TpNACs and other genes, with the TpNACs in yellow and other genes in blue.2.8 Homology modeling

We constructed a protein interaction network for TpNAC TFs and identified interactions of TpNAC protein with other TpNAC protein as well as non-TpNAC protein (Fig. 8). TpNAC20, TpNAC15 and TpNAC5 were in the regulatory center, and TpNAC20 interacted with 17 non-TpNAC protein. TpNAC15 was in the regulatory center of TpNAC protein and interacted with TpNAC7, TpNAC21, TpNAC48 and TpNAC58. TpNAC5 not only interacted with 4 other proteins but also formed a regulatory chain with TpNAC16, TpNAC47 and TpNAC32.

### Homology modeling

**Fig. 9:**
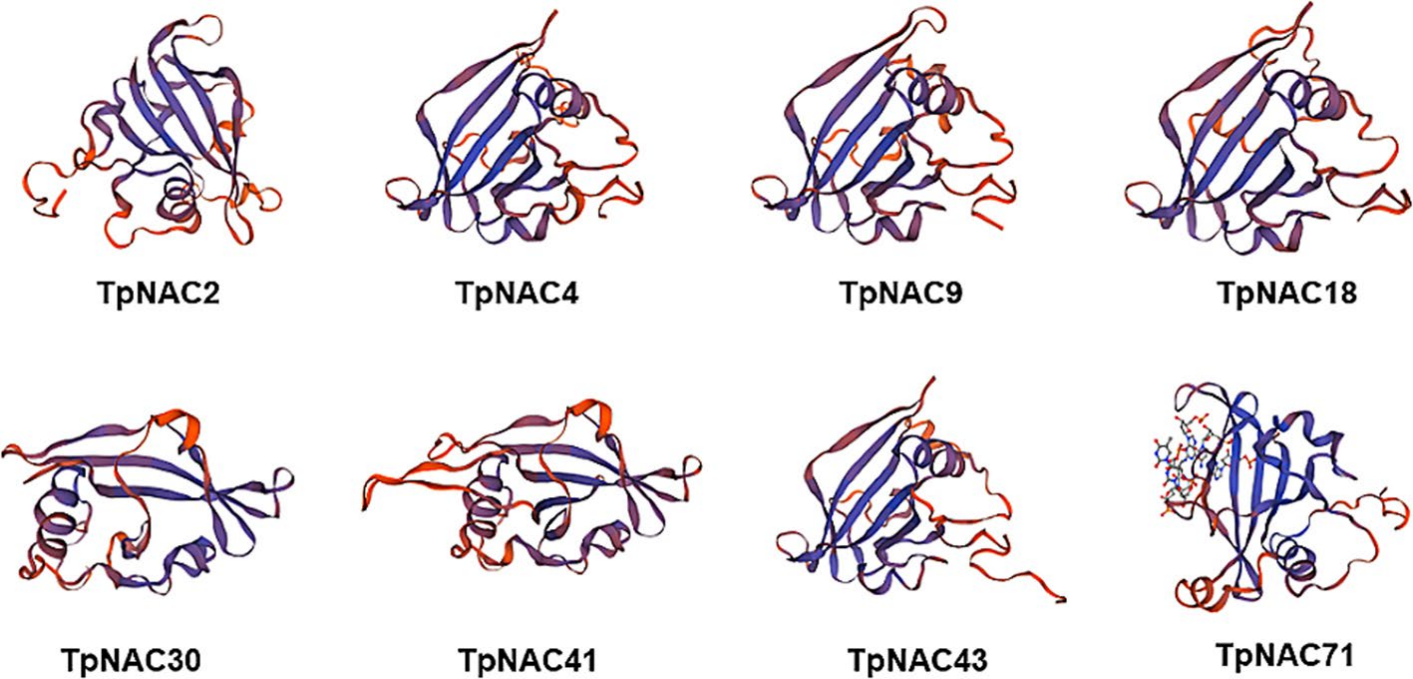
3D structure modeling of the TpNAC protein

**Fig. 10:**
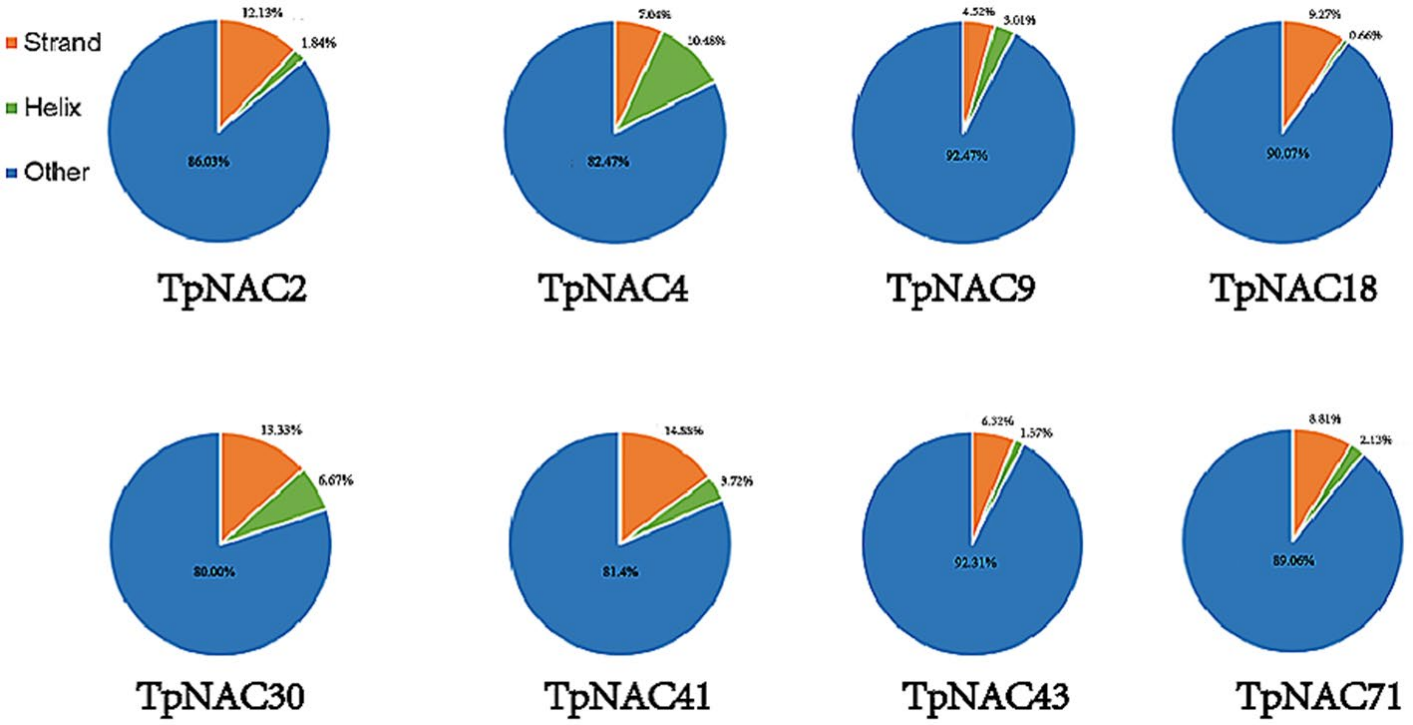
Secondary structure analysis of the TpNAC protein, with folds, helices, and other structures represented by different colors

The 3D structure of the TpNAC protein was predicted by homology modeling. PSI-BLAST results showed that the TpNAC protein could be divided into four configurations: 4dul, 3ulx, 3swp and 6gl3. However, the TpNAC proteins that were eventually successfully modeled belonged to 4dul (represented by TpNAC2, TpNAC4, TpNAC9, TpNAC18, TpNAC43), 3ulx (represented by TpNA30, CTpNAC41), and 3swp (represented by TpNAC71), with the largest number belonging to 4dul. The three-dimensional structure of the TpNAC proteins differed slightly among subfamilies. All three types of models had double-sided continuous α-helical structures, and the configuration was an intermediate continuous β-folded structure (Fig. 9). Secondary structure analysis showed that the proportion of β-folded TpNAC protein was higher than that of α-folded protein, except that the α helices accounted for a higher proportion of the total secondary structure than β-folds, and all the proteins were dominated by random curling (Fig. 10). In conclusion, the construction of the homology model of the TpNAC protein lays a foundation for further understanding the molecular function of TpNAC.

## Discussion

NAC TFs are important plant-specific TFs that are widely involved in plant responses to biological and abiotic stresses. Genome-wide NAC TF identification studies have been conducted in *Vigna radiata* [26], *Lolium perenne* [27], *Nelumbo nucifera* [28] and other plants. A high-quality genome sequence of red clover has been published, laying a foundation for genome-wide identification and analysis of NAC gene family members in red clover. In this study, a total of 72 *TpNAC* genes were identified in red clover, which was the same as the number of *NAC* genes identified in *Lolium perenne* [27] and less than the 105 *NAC* genes identified in *A. thaliana* [21], 152 in *G. max* [22], 93 in tomato [20], 132 in *Arachis hypogaea* [29], 81 in *Vigna radiata* [26], and 82 in *Nelumbo nucifera* [28]. It is possible that in the process of *TpNAC* amplification, some *TpNACs* related to signal transduction were preferentially preserved under selection pressure, forming the present TpNAC gene family. Similar to the results of studies in *O. sativa* [30], *L. esculentum* [31], *Nicotiana tabacum* [32] and *Juglans regia* [33], TpNACs exhibited large differences in physical and chemical properties and uneven chromosomal chromosomes but relatively conserved gene and protein structures. More than 50% of the *TpNAC* genes contained 3 exons, and only one *TpNAC* gene contained only one exon. A total of 10 motifs of the TpNAC gene family were identified, and the major conserved motifs of TpNACs were the same, which was similar to the results of Dactylis glomerata [34]. According to the research results for alfalfa (*Medicago sativa*), the 72 *TpNAC* genes were divided into 13 subfamilies. The *TpNAC* gene structure and motif distribution were similar withing the same subfamily, indicating that *TpNAC* genes in the same subfamily originated from the same ancestor and that genes from a common ancestor evolved independently at the same rate with little change.

Collinearity analysis showed that the *TpNACs* had 5 pairs of large fragment replicators distributed in subfamilies TERN, NAM and OsNAC7, as also observed in *Asparagus officinalis* [20] and fewer than the 15 pairs in *N. nucifera* [28], 116 pairs in *A. hypogaea* [35], and 17 pairs in *L. esculentum* [31]. Only a few tandem repeats were found in the above species, while no tandem repeats were found in *TpNACs*, suggesting that large segment replication is the main method of *NAC* gene amplification. Through interspecies collinearity analysis, 43, 37 and 26 pairs of *TpNAC* homologous genes were found in *M. truncatula*, *G. max* and *A. thaliana*, respectively, and the *TpNAC* homologs in *M. truncatula* were the most numerous. Interestingly, the number of *TpNAC* homologs in *G. max*, which is also a legume, was less than that in *A. thaliana*, a cruciferous plant.

Ten, six, five, two and five *NAC* genes related to drought tolerance were identified in chickpea [36], cauda [37], groundnut [29], poplar (*Populus tomentosa*) [38] and potato (*Dioscorea esculent*) [39], respectively. In *A. thaliana*, *ANAC019* improves the drought resistance of plants by regulating the expression of *DREB2A* and its downstream genes as well as key drought resistance genes such as *ARF2* and *DREB2A*. *ANAC072* (*RD26*) is involved in the regulation of drought resistance and salt tolerance in plants by ABA [40, 41]. Overexpression of *ATAF1* (*ANAC002*) in rice improved salt tolerance, and overexpression of *StNAC053* in sunflower improved the salt tolerance and drought tolerance of the plants [42–44]. This suggests that the NAC TF family is involved in plant responses to various biological stresses; at the same time, many cis-acting elements related to hormone and abiotic stress responses, such as AuxxRR-core, ABREs, CCAAT-box, CGTCA-motif, and TGACG-motif, were identified on the *TpNAC* gene promoter [45–49]. These results indicate that *TpNACs* play an important role in the mechanism of abiotic stress resistance in red clover.

Protein interaction network prediction can be used to predict gene function to some extent. The prediction results for the TpNAC protein interaction network showed that TpNACs were also associated with pollen development proteins such as VPS15 [50], VPS34 [51], MYB80 [52] and PIP5K4 [53], except under biological stress. WEE11 [54], PIS1 [55], JMJ14 [56], STM [57] and other proteins related to plant growth and development interact with genes related to plant immunity, such as PLC2 [58], indicating that TpNACs not only play an important role in plant resistance to abiotic stress but also play an important role in plant growth and development and resistance to biological stress.

To explore the role of the TpNAC TF family in resistance to lead stress in red clover, we determined the expression levels of 72 *TpNAC* genes under different concentrations of lead stress based on the RNA-seq data of Meng et al. [25]. All *TpNAC* genes were found to have different responses to lead stress. Among them, the expression levels of 5 *TpNAC* genes were upregulated, and those of 9 *TpNAC* genes were downregulated (Fig. 7). Eight *TpNAC* genes were selected for qRT‒PCR verification. *TpNAC29* and *TpNAC42* were found to belong to the NAC1 and NAM subfamilies, respectively, and the expression of these *TpNAC* genes was steadily downregulated under lead stress. In other species, the *NAC1* gene has been found to be widely involved in plant resistance to abiotic and biological stresses, and the *SNAC1* gene is overexpressed in rice, wheat, barley, cotton, maize, banana, or oat can improve drought resistance and salt tolerance [59]. Overexpression of *ZmSNAC1* in *Arabidopsis thaliana* increased sensitivity to abscisic acid (ABA) and osmotic stress during germination but increased tolerance to dehydration compared with that in wild-type plants [60]. SlNAC1 is a key transcription factor involved in plant defense mechanisms and positively regulates tomato resistance to *Pseudomonas* bacteria [61]. NAM subfamily members play important roles in plant resistance to abiotic and biological stresses. Liu et al. reported that 40% of NAM subfamily members in the NAC gene family of Liriodendron decreased significantly after being subjected to high-temperature stress at 40 °C [62]. The *CpNAC68* gene in wintersweet belongs to the NAM subfamily. Lin et al. overexpressed the *CpNAC68* gene in *Arabidopsis thaliana*, which enhanced the tolerance of the transgenic plants to cold, heat, salt, and osmotic stress [63]. In wheat, the NAM subfamily member *TaNAC21/22* has been shown to negatively regulate wheat resistance to stripe rust [64]. This study indicate that *TpNAC* genes are widely involved in the response of red clover to lead stress and that *TpNAC29* and *TpNAC42* have a common ancestor and play an important role in the resistance of red clover to lead stress. The results of this study will be helpful for further exploring the molecular processes underlying the role of the *TpNAC* genes in the resistance of red clover to lead stress and provide new insights for exploring the resistance of this plant to abiotic stress.

## Conclusion

In this study, the NAC gene family of *T. pratense* was identified for the first time, and chromosome mapping, phylogenetic analysis, collinearity analysis, motif and gene structure analysis, homeopathic element analysis, Pb stress expression pattern analysis and protein interaction network analysis were performed. A total of 72 *TpNAC* genes were identified and divided into subfamilies, which provided necessary information for the functional identification of *TpNAC* genes in *T. pratense*. The results of collinearity analysis showed that large fragment replication only existed in the TERN, NAM and OsNAC7 subfamilies and that *T. pratense* is closely related to *M. truncatula*. Interestingly, the number of *T. pratense* TpNAC homologs in *A. thaliana* was higher than that in *G. max*, another legume. Furthermore, 11 genes that may have played an important role in the evolution of the NAC gene family were identified. The expression pattern analysis showed that *TpNAC29* and *TpNAC42* had the most evident response to lead stress. The expression levels of *TpNAC29* and *TpNAC42* were significantly reduced after lead stress and were maintained at a level far lower than that without lead stress. The predicted protein interaction network indicated that TpNAC20, TpNAC15 and TpNAC5 may be in the center of their respective regulatory networks. Three-dimensional structure analysis showed that the TpNAC protein can be divided into the 4dul, 3ulx, and 3swp configurations, and the secondary structures of all the proteins was dominated by random curling. This study will be helpful for further studying the physiological and molecular processes of the *NAC* gene-mediated response to Pb stress in *T. pratense*, as well as the evolutionary process of *NAC* genes, and provides a basis for further understanding the NAC gene family and its mechanism of action in *T. pratense*.

## Materials and methods

### Identification of NAC TFs and chromosomal locations in *Trifolium pratense*

The genome sequence of red clover was downloaded from Phytozome (https://phytozome-next.jgi.doe.gov/), the HMM file of the NAM domain (PF02365) was retrieved from the Pfam database (http://pfam.xfam.org/), and NAC family proteins with e-values less than 0.001 were identified in the T. pratense database by HMMER 3.0. Partially incomplete and redundant amino acid sequences were deleted, and possible *TpNACs* were identified by Pfam and NCBI-CDD. The amino acid number, molecular weight (MW), theoretical isoelectric point (pI), instability coefficient, fat index and average hydrophilicity of each TpNAC protein sequence were calculated by ExPASy3. The protein sequences were submitted to CELLO (http://cello.life.nctu.edu.tw/) for subcellular localization prediction.

### Sequence alignment and phylogenetic and collinearity analyses

MEGA7 was used to compare the 72 TpNAC proteins identified, and the phylogenetic tree with the bootstrap value set to 1000 was constructed by the neighbor-joining algorithm. The whole-genome sequence of *T. pratense* was compared using local BLAST software, collinearity analysis was performed using MCScanX software, and gene replication was mapped using Circos software.

Related data for *A. thaliana*, soybean and *M. truncatula* were downloaded from Phytozome, and collinearity analysis between species was performed using MCScanX Python.

### Gene structure and conserved motif analysis

The motif analysis tool MEME5.4.1 was used to analyze the conserved motifs of the NAC gene family. The parameter was set to default, and the number of motifs was set to a maximum of 10. The gene structure information of the *TpNAC* gene was extracted from the annotated *T. pratense* gene structure file. TBtools software was used to visualize the relationships between the genes.

### Promoter cis‑acting element analysis

TBtools software was used to extract the 2 kb sequences upstream from the *TpNAC* gene transcription start sites from the *T. pratense* genome, and these were submitted to the PlantCARE tool for promoter analysis (http://bioinformatics.psb.ugent/webtools/plantcare/html/). TBtools was used to draw the distribution of components on each promoter.

### Transcriptomic resources and expression heatmap drawing

The transcriptome data were derived from a study published by Meng et al. [25], in which red clover was grown in soils with lead concentrations of 0, 500, 1000, 2000, and 3000 mg/kg for 45 days, and transcriptome and metabolome analyses were performed on the upper part of the collection site. Based on the FPKM values obtained in the study, TBtools was used to construct the expression heatmap.

### Plant materials and stress treatments

Healthy *T. pratense* seeds were sterilized with 10% NaClO for 10 min, rinsed with sterile water for 40 min to remove residual NaClO, soaked in sterile water for 2 min, and finally placed on a wet petri dish (25 °C) for germination for one week. Seedlings with uniform growth were selected and planted in soil to 45 days of age. *T. pratense* leaves with constant growth were transferred to soil with 1000 mg/kg Pb (NO_3_)_2_ and passivated for 2 weeks for lead stress treatment. Five plants were set per pot, and 3 groups of replicates were established. Leaf samples were collected at 0 h, 12 h, 24 h, 36 and 48 h, rapidly frozen in liquid nitrogen and stored at − 80 °C for subsequent qRT‒PCR. During the whole growth process, *T. pratense* leaves were exposed to 25 ± 1 °C with a 12 h/12 h light/dark cycle, and the leachate was returned to the basin.

### Total RNA isolation and qRT‒PCR expression analysis

Total RNA was extracted using a kit. TRIzol® was used for purification of RNA. DNase I was used for DNA removal. A NanoDrop ND-2000 spectrophotometer was used to determine RNA concentration, purity, and integrity, followed by 1% agarose gel electrophoresis to detect RNA quality. Reverse transcription was performed using Vazyme’s HiScript® III 1st Strand cDNA Synthesis Kit (+ gDNA wiper). Beacon Designer 7.9 software was used to design specific primers, and qRT‒PCR was used to verify the expression of the TpNAC gene using a Vazyme qRT‒PCR kit in a 10 µl mixture containing 5 µl of 2×ChamQ SYBR Color qPCR Master Mix (High ROX Premixed), 0.5 µl of cDNA product, 0.5 µl of each primer and 4.1 µl of ddH_2_O. The following qRT‒PCR scheme was used: one cycle of predenaturation at 95 °C for 30 s, one cycle, followed by 40 cycles of denaturation at 95 °C for 10 s and annealing/extension at 60 °C for 30 s. The 2^−∆∆Ct^ method was used to calculate fold changes in gene expression, and IBM SPSS Statistics 26 software was used to analyze the data. Plots were generated using Origin 2019b software.

### Co‑expression network construction

Total RNA was extracted using an Ultrapure RNA Kit (CWBIO, Taizhou, China). cDNA for reverse transcription PCR was synthesized using HiScript II Reverse Transcriptase (Vazyme, Nanjing, China). With cDNA used as a template, real-time fluorescence quantification was performed using internal reference and fluorescence quantification primers **(Table S1)**. All qRT–PCR analyses were performed using a ChanQ Universal SYBR qPCR Master Mix Kit according to the manufacturer’s instructions, and the relative gene expression was calculated using the 2^−∆∆Ct^ method.

### Homology modeling of the TpNAC 3D structure

In this paper, homology modeling was used to predict the three-dimensional structure of the TpNAC proteins. First, all protein model libraries were downloaded from PDB (http://www.rcsb.org/), and then the PSI-BLAST method was used to search for protein templates with the highest homology among TpNAC gene family members. TpNAC protein sequences with the corresponding template SWISS-MODEL (https://swissmodel.expasy.org/interacti/e) were used to predict the TaNAC protein 3D structure. Finally, SAVES (http://nihserver.mbi.ucla.edu/SAVES/) was used to detect the quality of the TpNAC protein three-dimensional structure.

## Data Availability

The data involved in this study are listed in the article and its additional files.
